# The effect of body mass index on the development of acute kidney injury and mortality in intensive care unit: is obesity paradox valid?

**DOI:** 10.1080/0886022X.2021.1901738

**Published:** 2021-03-22

**Authors:** Mehmet Süleyman Sabaz, Sinan Aşar, Gökhan Sertçakacılar, Nagihan Sabaz, Zafer Çukurova, Gülsüm Oya Hergünsel

**Affiliations:** a Department of Anesthesiology and Reanimation, Marmara University Pendik Training and Research Hospital, Istanbul, Turkey; b Department of Anesthesiology and Reanimation, Health Sciences University, Bakırköy Dr Sadi Konuk Training and Research Hospital, Istanbul, Turkey; c Division of Nursing, Department of Pediatric Nursing, Faculty of Health Sciences, Marmara University, Istanbul, Turkey

**Keywords:** Acute kidney injury, body mass index, obesity, mortality, intensive care unit, obesity paradox, mechanical ventilation

## Abstract

**Background:**

The conflicting results of studies on intensive care unit (ICU) mortality of obese patients and obese patients with acute kidney injury (AKI) reveal a paradox within a paradox. The aim of this study was to determine the effects of body mass index and obesity on AKI development and ICU mortality.

**Methods:**

The 4,459 patients treated between January 2015 and December 2019 in the ICU at a Tertiary Care Center in Turkey were analyzed retrospectively.

**Results:**

AKI developed more in obese patients with 69.8% (620). AKI development rates were similar in normal-weight (65.1%; 1172) and overweight patients (64.9%; 1149). The development of AKI in patients who presented with cerebrovascular diseases was higher in obese patients (81; 76.4%) than in normal-weight (158; 62.7%) and overweight (174; 60.8%) patients (*p* < 0.05). The risk of developing AKI was approximately 1.4 times (CI 95% = 1.177–1.662) higher in obese patients than in normal-weight patients. Dialysis was used more frequently in obese patients (24.3%, *p* < 0.001), who stayed longer in the ICU (*p* < 0.05). It was determined that the development of AKI in normal-weight and overweight patients increased mortality (*p* < 0.001) and that there was not a difference in mortality rates between obese patients with and without AKI.

**Conclusion:**

The risk of AKI development was higher in obese patients but not in those who were in serious conditions. Another paradox was that the development of AKI was associated with a higher mortality rate in normal-weight and overweight patients, but not in obese patients. Cerebrovascular diseases as a cause of admission pose additional risks for AKI.

## Introduction

Overweight and obesity occur as a result of the increase in the amount of body-fat to a degree that can negatively affect health [1]. The prevalence of obesity among adults is increasing significantly [2,3]. Obesity is recognized as an important public health problem. It has been shown to be associated with increased morbidity and mortality risks due to cardiovascular diseases, diabetes mellitus, respiratory tract diseases, some types of cancer, and all causes [2–4].

In parallel with the increase in obesity prevalence, there has been an increase in the number of obese patients admitted to intensive care units (ICUs) in recent years [5]. Previous studies have revealed that the rate of patients with a body mass index (BMI) of ≥30 in ICUs is between 17.5 and 28.0%, and nearly 7% have a BMI of ≥40 [6–9]. Obesity can be the cause of disorders in cardiovascular, respiratory, and metabolic functions in the ICU. Depending on the lipophilicity of the molecule administered, drug administration can be implemented. The capability to obtain vascular access is often damaged as a result of large body habitus, and it ought to be supported by ultrasound guidance. The quality of blood pressure monitoring can also be negatively influenced, requiring the utilization of direct intraarterial monitoring [10]. There is a strong relationship between obesity and ICU length of stay [7–10]. However, studies on the mortality impact of obesity on critically ill patients have yielded contradictory results [7,11–14]. Some studies have reported an increase in mortality [11–13], and, in the same way, other studies [7,14] have disclosed that obesity correlated with a longer period of mechanical ventilation and ICU length of stay; however, there is no association between mortality and obesity. Some studies stated that due to the fact that a high BMI provides nutritional reserves, exhibits protective hemodynamic effects of hypertension, which is common in obese patients, and, during circulatory failure, reduces the need for fluid or vasopressor support and the increased use of heparin prophylaxis among obese patients, effects such as inhibiting systemic inflammatory responses and coagulation phenomena have revealed that the obesity paradox is not harmful to patients but can be protective [15,16].

Another paradox associated with obesity is the development of acute kidney injury (AKI). Hospital-acquired AKI is a multifaceted and potentially reversible syndrome. Using the current diagnostic and grading criteria (increased serum creatinine levels and decreased urination), it was observed that more than 50% of ICU patients developed AKI. Renal replacement therapy is applied to 10–15% of ICU patients with severe AKI. AKI in obese patients includes all AKI stages from tubular dysfunction with mild to moderate increases in serum creatinine concentrations, to anuric acute renal failure requiring RRT [17]. Some of the previous studies that researched the relationship between AKI and mortality in obese patients followed in the ICU have reported conflicting results [18–24].

In addition to the complex results described regarding the ICU mortality of obese patients, the conflicting results of studies on obese patients who develop AKI reveal a paradox within a paradox. Our study was planned to evaluate the effects of BMI and obesity on AKI development and mortality in a large, single-center sample to elucidate this paradox.

## Materials and methods

### Data extraction center

This retrospective cohort study was conducted in the ICU of a training and research hospital with a bed capacity of 652, in Istanbul, Turkey. The ICU, which consists of 27 patient beds, accepts an average of 1440 medical, surgical, or trauma patients per year. The nurse-patient ratio is 1:2 in this center, which provides IC care as a closed unit controlled by a clinical decision support system (CDSS), where extracorporeal treatments (extracorporeal membrane oxygenation, hemodialysis, plasmapheresis) can be applied 24 h a day 7 days a week by intensive care (IC) specialists, IC minor assistants, and Anesthesiology and Reanimation specialists and assistants.

When a patient is admitted to the ICU, after removing the clothes and jewelry on the patient, height and weight are measured and recorded in the CDSS by the nurse. Treatments such as intravenous fluid and diuretics administered before the patient's admission are not considered during this measurement. After the weight and height information is entered into the system, the system automatically calculates and saves the BMI. BMIs of the patients are evaluated only when they are admitted to the ICU. The patient's urine output is monitored hourly and noted in the CDSS. In addition, the results of laboratory tests such as creatine requested during follow-up are automatically uploaded to the system. The CDSS monitors hourly urine output and creatine values by using data with AKI algorithm prepared according to Kidney Disease: Improving Global Outcomes (KDIGO) (Clinical Practice Guideline for Acute Kidney Injury) [25] criteria. If the AKI criteria defined in KDIGO are met by following the increases in creatine and the decreases in urinary output, the CDSS generates an alert and determines the stage. The system records the AKI alert and warns the user with an alarm. In this way, the development of AKI is detected immediately and precisely in the patients followed in the ICU.

In all patients who develop AKI according to the KDIGO criteria, nephrotoxic drugs are removed from the treatment, or if the treatment cannot be halted, the dose is modified consistent with glomerular filtration rate (GFR). The fluid status of the patient is assessed with dynamic tests, and suitable fluid treatment is regulated. The potential factors that could instigate the development of AKI are eradicated. The interventions were introduced in a protocolized manner (Furosemide Stress Test, Gambro and Kalmar Hospital protocols). Diuretics were administered to facilitate ventilation protocolized in oliguric patients with acute kidney injury when fluid overload and consequently respiratory parameters were affected. Renal replacement therapy (RRT) was initiated immediately upon the observation of life-threatening changes in fluid-electrolyte and acid-base equilibrium such as metabolic acidosis (potential hydrogen (pH) <7.15), hyperkalemia (potassium >6.5 mEq/l or ECG changes), anuria (urine output <100 ml/24 h), respiratory failure causing pulmonary edema, uremia with altered mental status (BUN >100 mg/dl). In addition, for solute control, fluid elimination, and correction of acid–base abnormalities, renal replacement therapy was introduced and implemented as continuous venovenous hemodiafiltration or continuous venovenous hemodialysis. Citrate was first used as an anticoagulant (based on Gambro and Kalmar Hospital protocols). The heparin was administered to patients who were not suitable for the use of citrate for anticoagulation. Furthermore, in the AKI algorithm in the CDSS, if the patient has a chronic renal failure (CRF) and is receiving RRT, this information is recorded in the relevant area in the CDSS so that the CDSS does not generate an AKI alert in these patients. Therefore, the system does not generate AKI alerts for the patients with CRF and for those receiving RRT. AKI data were acquired after these patients were excluded.

### Data collection

The data of patients followed up between 1 January 2015 and 31 December 2019 were obtained by EMRall-Qlin ICU Imd Soft Metavision CDSS with Structured Query Language (SQL) queries used in ICUs, and they were evaluated retrospectively. According to the anonymized age, gender, height, weight, BMI, diagnosis of comorbid diseases, ICU length of stay, and AKI alerts and AKI stages developed during the ICU stay; Acute Physiology and Chronic Health Evaluation II (APACHE II) and Simplified Acute Physiology Score III (SAPS III) scores measured in the ICU within the first 24 h were evaluated.

Mean Sequential Organ Failure Assessment score (SOFA) values calculated in the ICU, mean pH values obtained from blood gas samples taken in the ICU, partial pressure of oxygen (pO2) values, partial pressure of carbon dioxide (pCO2) values, bicarbonate anion (HCO_3_^−^) and lactate values, treatments (vasoactive agent, antibiotic) and interventions (arterial catheter, central venous catheters [CVC], mechanical ventilation, tracheostomy, hemodialysis) applied in the ICU, one of the mechanical ventilation parameters FiO2 (Fraction of inspired oxygen), minute respiratory rate, positive end-expiratory pressure (PEEP), P peak, tidal volume, compliance and work of breathing provided by a ventilator (WOBv) data, mechanical ventilation duration, and mortality data were evaluated.

### Study population

During the study period, a total of 7227 patients were admitted to the ICU. Four thousand four hundred fifty-nine patients who remained after the patients were excluded according to the exclusion criteria constituted the first study population.

According to the criteria of the World Health Organization [26], patients were classified according to their BMI. Eighty-seven patients with a BMI of <18.5 (underweight), 1801 patients with a BMI between 18.5 and 24.9 (normal weight), 1770 patients with a BMI between 25 and 29.9 (pre-obesity), 566 patients with a BMI between 30 and 34.9 (obesity class I), 187 patients with a BMI between 35 and 39.9 (obesity class II), and 135 patients with a BMI of ≥ 40 (obesity class III) were determined. Eighty-seven patients with a BMI of <18.5 were excluded from the study population to avoid large differences in sample sizes among the groups. In addition, the BMIs of all obese patients were combined into a single group of ≥30. Consequently, a total of 4459 patients were decided as the final study population, in which 1,801 patients had a BMI between 18.5 and 24.9 (normal weight), 1770 patients a BMI between 25 and 29.9 (overweight), and 888 patients a BMI of ≥30 (obesity).

### Inclusion criteria

All patients over the age of 18 and staying in the ICU longer than 24 h were planned to be included in the study.

### Exclusion criteria

For this study, we excluded patients who were younger than 18 years of age (*n*: 53), those who had a BMI of <18.5 (*n*: 87), those who were transferred to hospital wards within the first 24 h of ICU admission (*n*: 815), those with mortality within the first 24 h in the ICU (*n*: 621), and those who had missing data (*n*: 1192).

### Primary outcome

According to the BMI of patients followed in the ICU, the primary aim of the study was to evaluate the effect of obesity on AKI development in these patients.

### Secondary outcomes

Secondary objectives of the study were to compare patients’ BMI with comorbidities, admission diagnoses, scores calculated after admission to the ICU, laboratory values, interventions and treatments, mechanical ventilator data, AKI development and stages, and ICU mortality.

### Ethical issues

Before starting the research, institutional permission and ethics committee approval (Protocol code: 2020/232 -Decision number: 2020-08-06) was obtained from Bakırköy Dr. Sadi Konuk Training and Research Hospital Clinical Research Ethics Committee. The study conforms to the provisions of the 1995 Declaration of Helsinki (as revised in Brazil, 2013).

### Statistical analysis

The data collected in the study were evaluated with the SPSS 22.00 program (SPSS Inc., Chicago, IL). The Shapiro–Wilk test was used to check the normality of the data distribution. Categorical variables are given as frequency (*n*) and percentage (%), and numerical variables as mean ± standard deviation or median and interquartile intervals (IQR). One-way ANOVA test was used for the comparison of numerical data and Tukey test for *post hoc* analysis. When one-way ANOVA assumptions could not be achieved, the Kruskal–Wallis test was used, and the Mann–Whitney *U* test was used to determine the group that caused the difference. The Chi-square test was used in categorical variables, and Fisher's exact test was used when the conditions of the chi-square test were not met. A logistic regression model covering all patients was created in order to determine the differences in the risk of developing AKI between the groups determined according to BMI. It was thought that the severity of acute illness and chronic health conditions of the patients may be different and may affect the results. To avoid these differences and provide better randomization, patients were divided into four quarters according to their APACHE II scores (≤13, 13–20, 21–26, ≤27). Logistic regression models were created for the patients in each quarter to determine the differences in the risk of developing AKI between the groups determined according to BMI. Kaplan–Meier analysis was used to determine the effect of BMI on ICU mortality. A *p*-value of <0.05 was used to determine the significance.

## Results

A total of 4459 patients were analyzed. Forty-four percent of the study population were female, and the mean age was 60.08 ± 19.46. The mean BMI was determined as 27.05 ± 5.56 kg/m^2^ (min–max= 18.52–78.12). The most common comorbid disease was hypertension (1638; 36.7%), and the most common admission diagnosis was sepsis (991; 22.2%). The mean APACHE II score was 19.9 ± 8.9, the mean SAPS III score was 45.1 ± 14.2, and the mean SOFA score was 6 ± 4. Mechanical ventilation was applied to 3056 (68%) patients. The mean duration of ICU stay was 9.7 ± 13.8. AKI development was observed in 2941 (66%) patients. The mortality rate was 31.6%.

### Comparison of clinical features according to BMI

General characteristics of the patients according to BMI are given in Table 1. Of the 4459 patients, 1801 (40.4%) were normal-weight, 1170 (39.7%) overweight, and 888 (19.9%) obese. The mean age of obese patients was 64.69 ± 15.53, and it was higher than those of normal-weight and overweight patients (57.88 ± 21.53, 61.16 ± 18 years, respectively, *p* < 0.001). It was determined that the male gender was more common in normal-weight and overweight patients, while the female gender (630; 70.9%) was more common in obese patients (*p* < 0.001). Mean BMI values were 22.93 ± 1.62, 27.19 ± 1.30, and 35.44 ± 6.80 for normal-weight, overweight, and obese patients, respectively. Comorbid diseases were more prevalent in obese patients than in other groups (*p* < 0.001). The frequency of hypertension, diabetes mellitus, and COPD was higher in obese patients (*p* < 0.05). Cancer was more prevalent as a comorbid disease in normal-weight patients than in other groups (*p* < 0.05). Considering the patients’ admission diagnoses to the ICU, the most common admission diagnosis in all groups was sepsis. In the comparison between groups, sepsis (236; 26.2%) was more common in obese patients than in normal-weight and overweight patients (*p* < 0.001). The most common source of sepsis in obese patients was intra-abdominal sepsis (119; 13.4%). Other common diagnoses in obese patients were pulmonary diseases (pneumonia, COPD, and other causes) (146; 16.4%), cerebrovascular diseases (due to stroke and intracranial hemorrhage) (106; 11.9%), and postoperative follow-up (97; 10.9%). Cerebrovascular diseases were most prevalent in overweight patients (286; 16.2%), while trauma (224; 12.4%) and malignancy (151; 8.4%) were most prevalent in normal-weight patients (*p* < 0.05) (Table 1).

**Table 1.** Characteristics of patients according to BMI groups and admission diagnosis.

Parameters	Normal weight, *n*: 1801 (40.4%), *n* (%)	Overweight, *n*: 1770 (39.7%), *n* (%)	Obese, *n*: 888 (19.9%), *n* (%)	*p*-value
Body mass index (kg/m^2^) median (IQR)	23.37 (22.03–24.22)	27.34 (26.12–27.77)	33.20 (31.25–36.73)	
Age (year) mean ± SD	57.88 ± 21.53	61.16 ± 18.0^a^	64.69 ± 15.53^b,c^	<0.001
Gender				<0.001
Female	648 (36)	682 (38.5)^a^	630 (70.9)^b,c^	
Male	1153 (64)	1088 (61.5)	258 (29.1)	
Comorbidity	1265 (70.2)	1321 (74.6)^a^	752 (84.7)^b,c^	<0.001
Hypertension	495 (27.5)	662 (37.4)^a^	481 (54.2)^b,c^	<0.001
Diabetes	270 (15.0)	397 (22.4)^a^	326 (36.7)^b,c^	<0.001
Cerebrovascular disease	134 (7.4)	126 (7.1)	63 (7.1)	0.917
CAD	206 (11.4)	267 (15.1)^a^	121 (13.6)	0.006
COPD	157 (8.7)	181 (10.2)	126 (14.2)^b,c^	<0.001
CRF	147 (8.2)	157 (8.9)	99 (11.1)^b^	0.038
Malignancy	256 (14.2)^a,b^	211 (11.9)	92 (10.4)	0.011
Hepatic disease	32 (1.8)	28 (1.6)	18 (2)	0.706
Psychiatric disorder	43 (2.4)	36 (2)	13 (1.5)	0.283
Dementia	93 (%5.2)^a^	621 (%3.5)	34 (3.8)	0.038
Other	195 (10.8)	168 (9.5)	66 (7.4)^b^	0.019
Admission diagnosis				
Cerebrovascular disease	252 (14)	286 (16.2)^c^	106 (11.9)	0.011
Cardiac	89 (4.9)	102 (5.8)	58 (6.5)	0.220
Pulmonary	205 (11.4)	199 (11.2)	146 (16.4)^b,c^	<0.001
Pneumonia	130 (7.2)	120 (6.8)	65 (7.3)	0.830
COPD	48 (2.7)	56 (3.2)	53 (6)^b,c^	<0.001
Pulmonary, other	27 (1.5)	23 (1.3)	28 (3.2)^b,c^	0.002
Renal-metabolic	108 (6)	82 (4.6)	61 (6.9)^c^	0.042
Hepatic cirrhosis	19 (1.1)	20 (1.1)	12 (1.4)	0.792
Trauma	224 (12.4)^b^	208 (11.8)^c^	57 (6.4)	<0.001
Sepsis	383 (21.3)	373 (21.1)	236 (26.2)^b,c^	0.002
Pneumosepsis	91 (5.1)	77 (4.4)	38 (4.3)	0.524
İntra-abdominal sepsis	164 (9.1)	177 (10)	119 (13.4)^b,c^	0.002
Urosepsis	41 (2.3)	29 (1.6)	18 (2)	0.388
Sepsis, other	87 (4.8)	89 (5.0)	61 (6.9)^b^	0.067
Intoxication	87 (4.8)^a,b^	49 (2.8)	16 (1.8)	<0.001
Malignancy	151 (8.4)	134 (7.6)	46 (5.2)^b,c^	0.011
Postoperative	150 (8.3)	186 (10.5)^a^	97 (10.9)^b^	0.035
GIB-hemorrhage	53 (2.9)	57 (3.2)	18 (2)	0.215
Other	80 (4.4)	75 (4.2)	35 (3.9)	0.831

CAD: coronary artery disease; COPD: chronic obstructive pulmonary disease; CRF: chronic renal failure; GIB: gastrointestinal bleeding.

^a^Normal weight versus overweight *p* < 0.05.

^b^Normal weight versus obese *p* < 0.05.

^c^Overweight versus obese *p* < 0.05.

The AKI stages, interventions, and treatments are shown in Table 2. The number of arterial catheters applied to the patients was similar in all groups. More central venous catheters were used in obese patients (502, 56.5%, *p* < 0.05). There was no difference between the groups in terms of TPN and antibiotic and vasoactive agent use (*p* > 0.05). APACHE II scores were lower in normal-weight patients (24 [17–29]) than in overweight (25 [19–30]) and obese (26 [19–31]) patients (*p* < 0.05). The highest APACHE II scores were determined to be in obese patients (*p* < 0.05). SAPS III scores were higher in obese patients (48 [37–60]) than in overweight (50 [39–60]) and normal-weight (49 [41–61]) patients (*p* < 0.05). Obese patients’ SOFA scores (6 [3–9]) were higher (*p* < 0.05) than those of normal-weight patients (5 [3–9]) and similar to those of overweight patients (6 [3–9]) (Table 3).

**Table 2.** AKI stages, interventions, and treatments during the ICU stay.

Parameters	Normal weight, *n*: 1801 (40.4%), *n* (%)	Overweight, *n*: 1770 (39.7%), *n* (%)	Obese, *n*: 888 (19.9%), *n* (%)	*p*-value
AKI	1172 (65.1)	1149 (64.9)	620 (69.8)^b,c^	0.025
Stage 1	98 (5.4)	118 (6.7)	57 (6.4)	0.286
Stage 2	156 (8.7)	185 (10.4)	101 (11.4)	0.054
Stage 3	918 (51.0)	846 (47.8)	462 (52.0)	0.062
Percentage of stages in AKI patients				
Stage 1	98 (8.4)	118 (10.3)	57 (9.2)	0.284
Stage 2	156 (13.3)	185 (16.1)	101 (16.3)	0.105
Stage 3	918 (78.3)^a^	846 (73.6)	462 (74.5)	0.023
Interventions				
Arterial catheter	1224 (68)	1253 (70.8)	613 (69)	0.183
Central catheter	858 (47.6)	903 (51)^a^	502 (56.5)^b,c^	<0.001
MV	1191 (66.1)	1230 (69.5)^a^	635 (71.5)^b^	0.010
Tracheostomy	307 (17)	345 (19.5)	165 (18.6)	0.164
Dialysis	295 (16.4)	329 (18.6)	216 (24.3)^b,c^	<0.001
Treatments				
TPN	618 (34.3)	655 (37)	318 (35.8)	0.243
Antibiotics	1534 (85.2)	1506 (85.1)	773 (87)	0.346
Vasoactive agents	985 (54.7)	1005 (56.8)	520 (58.6)	0.142

AKI: acute kidney injury; MV: mechanic ventilation; TPN: total parenteral nutrition.

^a^Normal weight versus overweight *p* < 0.05.

^b^Normal weight versus obese *p* < 0.05.

^c^Overweight versus obese *p* < 0.05.

**Table 3.** The average of ICU scores, mechanical ventilator values, and blood parameters of the patients during ICU follow-up.

Parameters	Normal weight Median (IQR)	Overweight Median (IQR)	Obese Median (IQR)	*P*-value
APACHE 2	24 (17–29)	25 (19–30)^a^	26 (19–31)^b^	<0.001
SAPS 3	48 (37–60)	50 (39–60)	49 (41–61)^b,c^	<0.001
SOFA	5 (3–9)	6 (3–9)	6 (3–9)^b^	0.019
MV (day)	4.81 (2.74–11.14)	5.73 (2.51–12.16)	5.89 (2.75–12.47)	0.242
LOS ICU (day)	5.45 (3–12.72)	6.54 (2.84–13.58)^a^	6.81 (3.32–13.88)^b^	0.004
FiO_2_ (%)	42.2 (39.4–46.6)	42.2 (39.9–46.1)	42.6 (39.9–46.6)^b,c^	<0.001
PEEP (cmH_2_O)	5.4 (5.1–5.9)	5.4 (5.1–5.9)	5.6 (5.1–6.1)^b,c^	<0.001
P peak (cmH_2_O)	13.94 (12.13–15.86)	14.06 (12.64–15.87)^a^	15.17 (13.52–17.02)^b,c^	<0.001
Tidal volume (ml)	481 (427–541)	497 (442–569)^a,c^	476 (423–534)	<0.001
Tidal volume /IBW (ml/kg)	6.72 (5.98–7.58)	6.83 (6.08–7.68)^a^	7.05 (6.30–7.99)^b,c^	<0.001
Respiratory rate (min)	16.7 (14.8–19.6)	16.3 (14.7–19.1)	16.8 (14.7–19.2)	0.590
Compliance (ml/cm H_2_O)	41.2 (31.4–51.3)^b^	41 (32.8–51.3)^c^	35.5 (29.8–43.9)	<0.001
WOBv (j/l)	1.03 (0.91–1.20)	1.06 (0.92–1.21)^a^	1.17 (1–1.32)^b,c^	<0.001
Mechanical power (J/min)	9.35 (7.64–11.03)	9.37 (7.87–11.23)	9.61 (7.89–11.52)	0.066
pH	7.39 (7.33–7.43)^b^	7.39 (7.32–7.43)	7.38 (7.30–7.42)	0.002
pO_2_ (mmHg)	94.9 (72.3–118.4)^b^	95 (75.5–116.3)^c^	91.5 (71.3–107.4)	<0.001
pCO_2_ (mmHg)	42.2 (37.1–49.5)	42.9 (38.3–48.9)	44.8 (40.7–54.5)^b,c^	<0.001
HCO_3_ (mEq/l)	24.9 (21.6–28.7)	25.1 (21.7–28.1)	24.7 (21.1–29)	0.820
Lactate (mmol/l)	1.78 (1.32–3.05)	1.96 (1.46–3.19)	1.98 (1.43–3.44)	0.297
Glucose (mg/dl)	137 (118–163)	149 (125–178)^a^	157 (132–197)^b^	<0.001
Nutrition (kcal/day)	1795 (1560–2086)^b^	1763 (1539–2082)^c^	1615 (1442–1865)	<0.001

APACHE: acute physiology and chronic health evaluation; SAPS: simplified acute physiology; SOFA: sequential organ failure assessment; MV: mechanic ventilation; LOS: length of stay; FiO_2_: fraction of inspired oxygen; PEEP: positive end-expiratory pressure; IBW: ideal body weight; pCO_2_, partial pressure of carbon dioxide; pO_2_, partial pressure of oxygen; HCO_3_: bicarbonate; WOBv: work of breathing ventilator; IQR: inter quartile range.

^a^Normal weight versus overweight *p* < 0.05.

^b^Normal weight versus obese *p* < 0.05.

^c^Overweight versus obese *p* < 0.05.

### Mechanical ventilator and blood gas parameters

When the patients who received invasive mechanical ventilator support during the IC follow-up were examined, it was seen that the need for mechanical ventilators increased as the BMI increased. While 66.1% (1,191) of normal-weight patients needed mechanical ventilator support, this rate increased to 69.5% (1,230) in overweight patients and to 71.5% (635) in obese patients (*p* < 0.05). Considering the mechanical ventilator parameters, the values of FiO2 (42.6 [39.9–46.6)], PEEP (5.6 [5.1–6.1]), peak pressure (15.17 [13.52–17.02]), and WOBv (1.17 [1–1.32]) were higher in obese patients, while compliance (35.5 [29.8–43.9]) and tidal volumes (476 [423–534]) were lower. When tidal volume is analyzed in ml/kg according to ideal body weight, it was higher in obese patients (7.05 [6.30–7.99]) than in overweight (6.83 [6.08–7.68]) and normal-weight (6.72 [5.98–7.58]) patients (Table 3). Minute Respiratory Rate and duration of mechanical ventilation were higher in obese patients, but this difference was not statistically significant. When looking at blood gas parameters, pH and pO_2_ were lower in obese patients, while pCO_2_ was higher (*p* < 0.05). HCO_3_ and lactate levels were found similar among the groups (*p* > 0.05) (Table 3).

### Nutrition

Obese patients were given fewer daily calories than other groups (1615 [1442–1865]; *p* < 0.05). Despite the low-calorie input, the mean blood sugar was found to be higher in obese patients than in other groups (157 [132–197]; *p* < 0.05) (Table 3).

### AKI development and dialysis

**Figure 1.**
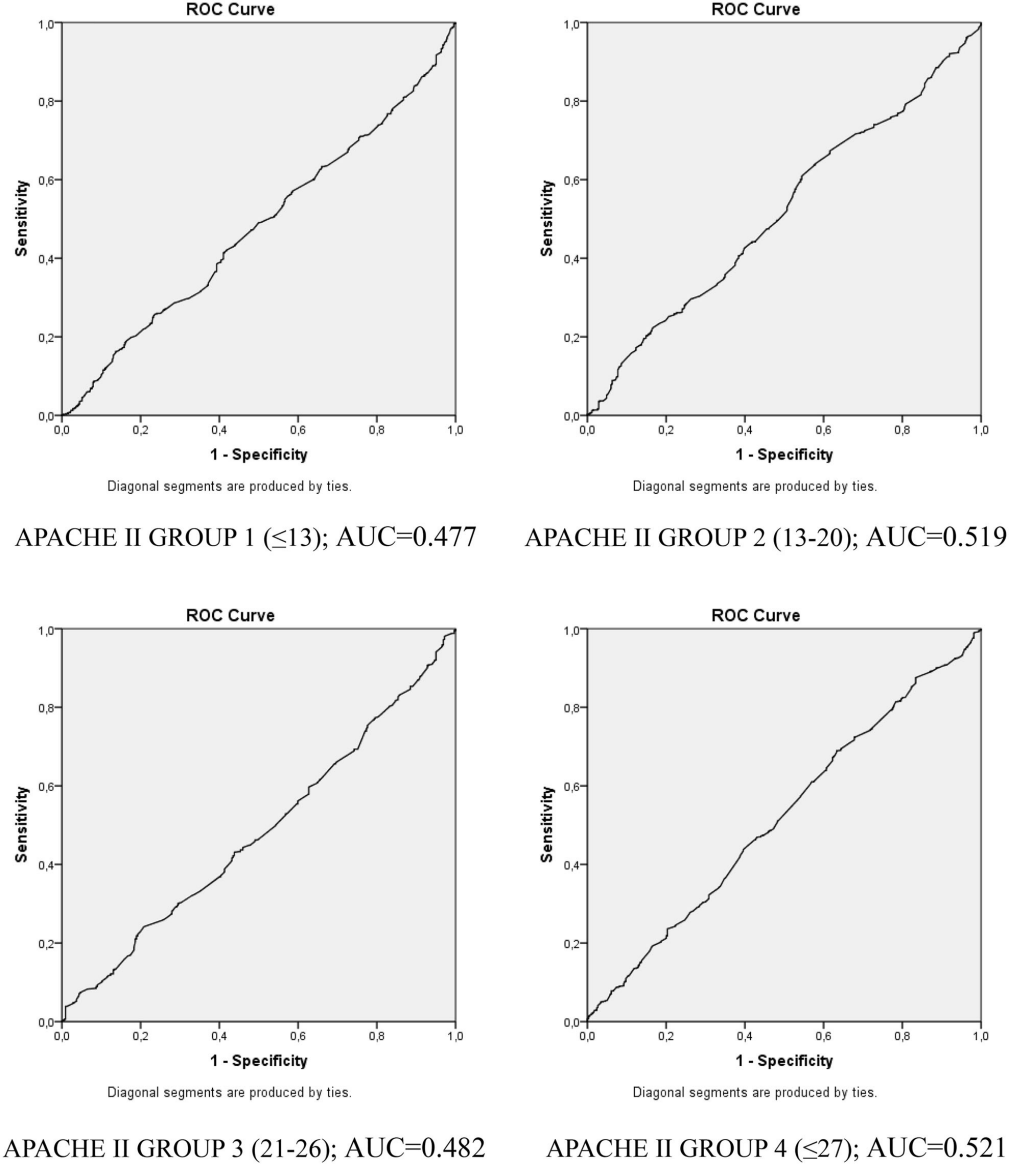
ROC analysis curves of BMI and AKI according to four different APACHE groups.

According to AKI alerts generated by the CDSS, most alerts were delivered for obese patients with 69.8% (620) (*p* < 0.05). The rate of AKI development was similar in normal-weight (65.1%; 1172) and overweight patients (64.9%; 1149). Regarding the stages of the patients who received an AKI alert, patients who received Stage 1 and Stage 2 AKI alert did not show a statistically significant difference among the groups, while the rate of Stage 3 AKI alert in normal-weight patients (918; 78.3%) was higher than that of overweight patients (46; 73.6%) (*p* < 0.05) and similar to that of obese patients (462; 74.5%) (Table 2). Logistic regression to determine the risk of AKI development according to patients' BMI revealed that the risk of developing AKI was approximately 1.4 times higher (OR: 1.398; CI95% = 1.177–1.662) in obese patients than in normal-weight patients. Considering that the clinical weights of the patients may differ, the disease severity was divided into four quarters according to the APACHE II group, and logistic regression was performed in patients with similar disease severity for the effect of BMI on the AKI development. The risk of developing AKI was found to be higher in obese patients in all groups. However, a statistically significant difference emerged in low APACHE II scores. Obese patients with an APACHE II score in the first quarter (≤13) had a 1.47 times higher risk of developing AKI (OR: 1.476 CI 95% = 1.054–2.068), and it was determined that the risk of developing AKI was 1.62 times higher in obese patients with an APACHE II score in the second quarter (13–20) (OR: 1.625 CI 95%=1.138–2.321) than in normal-weight patients in the same quarter. Patients with an APACHE II score in the third (21–26) and fourth quarters (≤27) showed no significant difference in terms of AKI development (Table 4). In the subgroup analysis performed according to the admission diagnosis, it was determined that the development of AKI in patients who were admitted with cerebrovascular diseases was higher in obese patients (81; 76.4%) than in normal-weight (158; 62.7%) and overweight (174; 60.8%) patients (*p* < 0.05). There was no statistically significant difference between the groups in terms of the development of AKI in other admission diagnoses (Table 5). When looking at the patients who developed a need for dialysis, obese patients (216; 24.3%) needed significantly more dialysis than normal-weight (295; 16.4%) and overweight (329; 18.6%) patients (*p* < 0.001). ROC analyses performed to determine cutoff values in the relationship of BMI with AKI development in different APACHE II groups did not yield significant results (Figure 1).

**Table 4.** Logistic regression of the risk of AKI development according to the BMI.

Group	*p*-value	OR	CI 95
All patients			
Normal weight	Reference		
Overweight	0.823	1.016	0.887–1.163
Obese	<0.001	1.398	1.177–1.662
APACHE II GROUP 1 (≤13)			
Normal weight	Reference		
Overweight	0.606	1.069	0.829–1.379
Obese	0.024	1.476	1.054–2.068
APACHE II GROUP 2 (13–20)			
Normal weight	Reference		
Overweight	0.246	1.179	0.893–1.559
Obese	0.008	1.625	1.138–2.321
APACHE II GROUP 3 (21–26)			
Normal weight	Reference		
Overweight	0.130	0.795	0.591–1.070
Obese	0.759	1.059	0.733–1.530
APACHE II GROUP 4 (≤27)			
Normal weight	Reference		
Overweight	0.650	1.065	0.811–1.398
Obese	0.114	1.309	0.938–1.826

APACHE: acute physiology and chronic health evaluation; OR: odds ratio; CI: confidence interval.

**Table 5.** AKI development according to the diagnosis of admission to the ICU and mortality rates to BMI groups.

Admission diagnosis	Normal weight, *n* (%)	Overweight *n* (%)	Obese, *n* (%)	*p*-value
Cerebrovascular disease	158 (62.7)	174 (60.8)	81 (76.4)^a,b^	0.014
Cardiac	55 (61.8)	63 (61.8)	41 (70.7)	0.465
Pulmonary	151 (73.7)	140 (70.4)	108 (74.0)	0.685
Pneumonia	99 (76.2)	88 (73.3)	49 (75.4)	0.872
COPD	34 (70.8)	39 (69.6)	37 (69.8)	0.990
Pulmonary, other	19 (67.9)	13 (56.5)	22 (78.6)	0.241
Renal-metabolic	51 (47.2)	50 (61)	32 (52.5)	0.170
Hepatic cirrhosis	133 (59.4)	126 (60.6)	37 (64.9)	0.747
Trauma	30 (56.6)	31 (54.4)	8 (44.4)	0.667
Sepsis	14 (73.7)	17 (85.0)	7 (58.3)	0.244
Pneumosepsis	291 (76)	272 (73.1)	173 (73.3)	0.619
Intra-abdominal sepsis	76 (83.5)	61 (79.2)	29 (76.3)	0.596
Urosepsis	131 (79.9)	130 (73.4)	99 (83.2)	0.113
Sepsis, other	22 (53.7)	14 (48.3)	6 (33.3)	0.354
Intoxication	50 (57.5)	45 (50.6)	37 (60.7)	0.322
Malignancy	36 (41.4)	23 (46.9)	10 (62.5)	0.286
Postoperative	101 (66.9)	89 (66.4)	32 (69.6)	0.924
GIB-hemorrhage	96 (64)	118 (63.4)	68 (70.1)	0.503
Other	57 (71.3)	46 (61.3)	23 (65.7)	0.425
Mortality				
All patient	548 (30.4)	556 (31.4)	307 (34.6)	0.091
AKI patient	388 (33.1)	399 (34.7)	223 (36.0)	0.198
AKI–mortality relationship				
AKI yes	388 (33.1)			<0.001
AKI no	160 (25.4)			
AKI yes		399 (34.7)		<0.001
AKI no		157 (25.3)		
AKI yes			223 (36.0)	0.183
AKI no			84 (31.3)	

COPD: chronic obstructive pulmonary disease; GIB: gastrointestinal bleeding; AKI: acute kidney injury.

^a^Normal weight versus obese *p* < 0.05.

^b^Overweight versus obese *p* < 0.05.

### AKI and mortality

**Figure 2.**
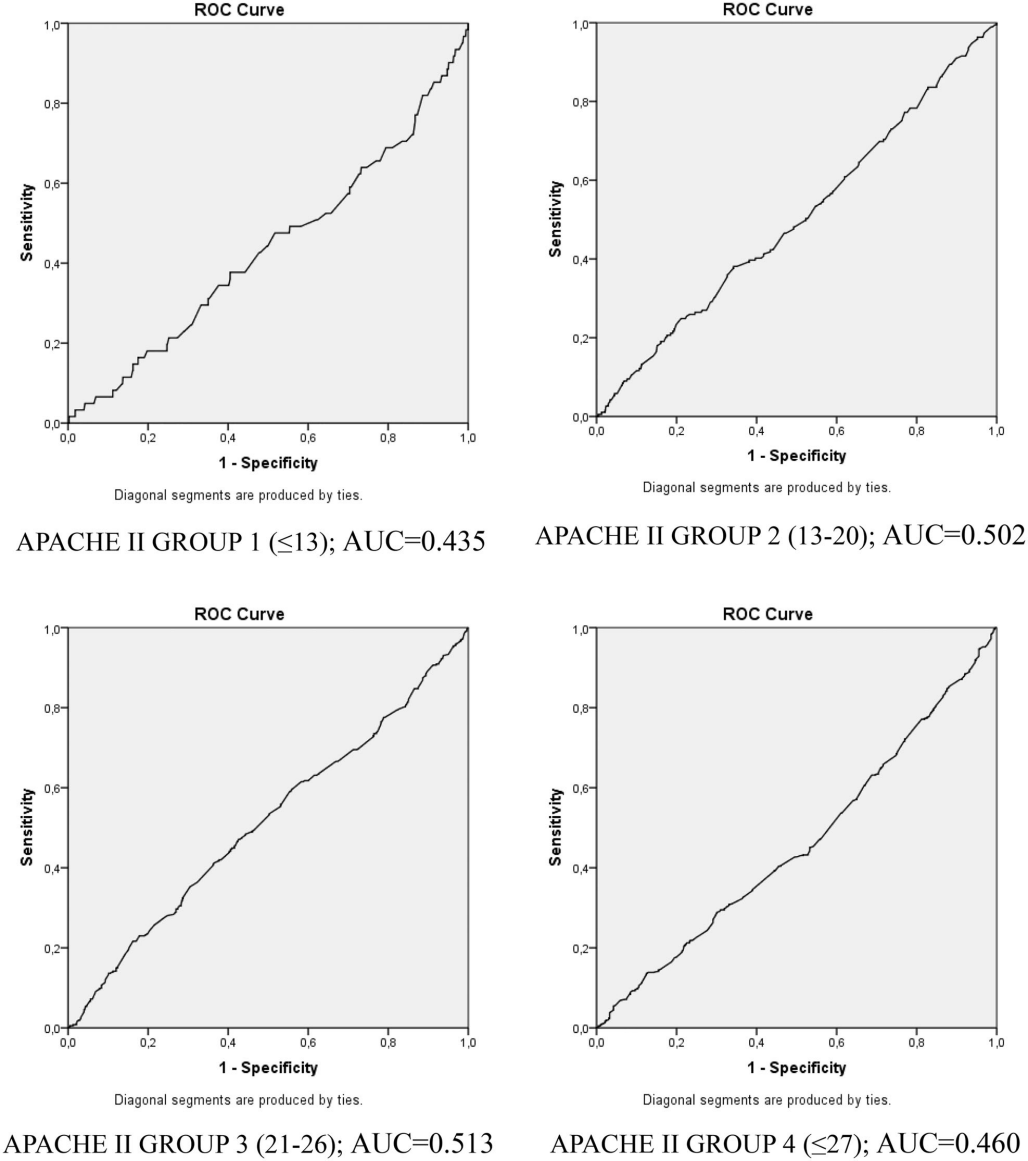
ROC analysis curves of BMI and mortality according to four different APACHE groups.

Patients who developed AKI needed more mechanical ventilator support in all three groups than patients without AKI and stayed longer in the ICU (*p* < 0.001). There was no difference in mortality between obese, normal-weight, and overweight patients who developed AKI. While it was determined that the development of AKI in normal-weight and overweight patients increased mortality (*p* < 0.001), there was no significant difference between the mortality of obese patients who developed AKI (223; 36.0%) and the mortality of obese patients without AKI (84;31.3%). The results of ROC analyses performed to determine cutoff values in the relationship of BMI with mortality in different APACHE II groups were not significant (Figure 2).

### ICU length of stay and mortality

Obese patients (6.81 [3.32–13.88]) had longer ICU length of stay (*p* < 0.05) than normal-weight (5.45 [3–12.72]) and overweight (6.54 [2.84–13.58]) patients. Mortality rates did not differ significantly among the groups. Even though the patients were grouped according to their admission diagnoses, there was no difference in mortality between any of the admission groups (*p* > 0.05) (Table 2). As a result of ROC analysis performed to determine the relationship between BMI and mortality, the area under the curve was found to be only 51.6% (0.516).

## Discussion

As a result of this study, which investigated the effect of BMI on AKI and mortality, it was determined that as the BMI increased, the rate of AKI development also increased. Especially obese ICU patients with low APACHE II scores were found to have a higher risk of developing AKI. Consistent with the results of our study, it has been shown in previous studies that the prevalence of AKI in the ICU is higher in obese patients than in non-obese, critically ill patients and that the prevalence of AKI in critically ill patients increases with BMI [17]. Danziger et al. [23] retrospectively evaluated the relationship between obesity, AKI, and AKI severity in 14,986 critically ill patients in the United States and found that obese patients developed more AKI than non-obese patients. Again, in a retrospective study conducted with 751 American ICU patients, Soto et al. [22] determined that obesity was associated with increased AKI development and that each 5 kg/m^2^ increase in BMI was associated with a 20% increased risk for developing AKI. In a large study conducted in Austria, it was determined that the risk of developing AKI increased more than two times in severely obese patients compared to normal-weight patients [18]. In a study conducted with 11,736 patients in Australia, Yap et al. found that obesity and morbid obesity were associated with an increased risk for AKI [27]. In a Danish study with 56,420 patients evaluating the effect of BMI on AKI risk retrospectively, Pedersen et al. reported that obese patients (BMI ≥30) showed an increased risk of developing AKI compared to patients with normal weight (BMI 18.5–24.9) [21].

Although the pathophysiology of increased AKI development in obesity is not fully understood, increased renal blood flow and GFR due to altered renal hemodynamics may increase the filtration fraction and increase the sensitivity to damage [28,29]. In addition to increased inflammatory mediators in response to acute diseases, increased production of leptin, an adipocyte-derived cytokine that controls energy metabolism and appetite and is metabolized in the kidney, and a significant decrease in the production of adiponectin, a hormone that regulates glucose and lipid metabolism, have been associated with an increased risk of AKI in obesity [30–32]. Obesity is associated with high HDL levels that bind bacterial lipopolysaccharide endotoxins [33]. In addition, autophagy, an important protection system in which damaged cells are eliminated, is impaired in obesity [34]. Obese ICU patients are at risk for increased intra-abdominal pressure, which may result in renal dysfunction due to both venous occlusion and poor arterial perfusion [35,36]. As determined in our study in obese patients, the effects of more common comorbid diseases on kidney physiology should not be ignored. Glance et al. [37] found that obese patients with metabolic syndrome faced a 3–7 times higher risk of AKI. Another factor that may contribute to the impact of obesity on AKI is the difficulty in assessing intravascular volume status and determining adequate fluid therapy or the dose of vasoactive agents.

In our study, mechanical ventilation durations of obese patients were found to be higher than those of normal-weight and overweight patients, but this difference was not statistically significant. Previous studies have revealed conflicting results for the impact of obesity on mechanical ventilation duration [6,16,38]. In a study [6], while the duration of mechanical ventilation was longer in obese patients than in non-obese patients, there was no difference in another similar study [16,38]. Obesity triggers changes in respiratory function, particularly a decline in respiratory capacity and an upsurge in airway resistance [39]. When looking at mechanical ventilator parameters, obese patients had higher PEEP and peak pressures as well as lower compliances. The ventilator workload increased. These results are consistent with previous research. During mechanical ventilation, obese patients are more inclined to atelectasis and require higher PEEP support to prevent it [40]. Moreover, studies have stated that obese patients require higher inspiratory pressure and plateau pressures [41,42]. Despite the exposure of higher pressures [43,44] on the mechanical ventilator that obese patients underwent, they tend to have a lower functional residual capacity and lung volume [45]. The lower compliance of obese patients can be explained by the reduction in the lung and chest wall compliance caused by obesity and making the lungs more susceptible to atelectasis or increased alveolar wall tension [46]. Abdominal visceral fat accumulation can increase the respiratory workload by causing an increase in intra-abdominal pressure [43,44]. In addition, studies have determined that obese patients consume more oxygen and produce more carbon dioxide [47,48]. In obese patients, increased respiratory workload and ventilation/perfusion mismatched as a result of early airway closure may clarify this situation [45]. In the present study, the ICU follow-up period was found to be significantly longer in obese patients than in non-obese patients. While some similar studies reported an increase in the ICU length of stay for obese patients [49,50], others could not find a difference between the ICU length of stay of obese patients and other patient groups [16,24,51]. This situation can be explained by the differences occurring among obese patients. There is no single obesity type in the ICU: obese patients vary according to their obesity class and comorbid disease burden. Also, some clinicians tend to think that obese patients have potentially worse outcomes [17]. Therefore, the increased acceptance of obese patients who can be treated more easily in the ICU and who are not very critical may shorten the follow-up period in the ICU. On the other hand, vascular access problems are common in obese patients, and the frequency of using central catheters is increased, as determined in our study. This may lead to infection development and catheter-related complications, prolonging the ICU stay. Nursing care of obese patients is more challenging due to their high body weight and large body surface area. Difficulty in changing the patient's position brings the risk of pressure sores and increased skin lacerations [52]. Deterioration of the skin barrier may increase complications such as infection or bleeding and might prolong the ICU stay.

### Mortality

In the present study, no difference was found between the groups in terms of ICU mortality. The phenomenon called the obesity paradox (associating obese patients with better survival in the ICU) could not be statistically confirmed. In addition, the ROC analysis performed to find a cutoff value where BMI affected mortality showed that determining such a cutoff value was almost indistinguishable from coin-tossing. However, despite the fact that obese patients had higher APACHE II scores, higher frequency of comorbid diseases, and higher ages in our study, the absence of a difference in mortality supports this paradox. In similar studies [14,53], the results on ICU mortality indicated that mortality was either lower in obese patients or was not any different [15,54,55]. In fact, Falagas et al. obtained higher mortality results [56] in a subgroup of obese patients. Even when the effect of the development of AKI [17] on ICU mortality in our study is considered, the development of AKI increased mortality in normal-weight and overweight patient groups, but interestingly, not in obese patients. The fact that mortality does not change even when the AKI table is added appears as another finding that supports the obesity paradox. Similar studies have obtained different results regarding the effect of AKI development on mortality in obese patients followed in the ICU. While some studies determined a decrease in mortality [18–20], some others did not find a difference in mortality [21–24]. Some studies determined that there was a U or J-shaped relationship between mortality and BMI [18,19]. In another study [57], while there was no difference in low disease scores, it was determined that higher disease scores differed in mortality and that obese patients had a survival advantage in higher disease scores. The timing of AKI and the importance of early diagnosis were emphasized in another study [58]. These complex results may have arisen due to the use of arbitrary obesity definitions (such as BMI> 28, BMI> 31) or due to differences in obese patient profiles and comorbid disease burdens. In addition, the fact that BMI measurement does not consider fat distribution and lean muscle mass may cause the misinterpretation of obesity. A patient with metabolic and clinical risks of obesity due to increased visceral fat mass cannot be classified as obese, while a patient with a high muscle mass and preserved visceral fat ratio can be classified as obese. Therefore, the BMI measurement used in obesity determination can also cause these contradictory results.

Our study has some strengths such as minimizing the data loss as a result of receiving our study data by electronic query by the CDSS and preventing human errors. However, it also has some limitations. First of all, having a single-center cohort prevents the results from being generalized. The retrospective design of the study may cause confounding factors affecting the results and may lead to the risk of bias. In addition, the fact that obesity is defined according to BMI and the absence of additional data to support, such as waist circumference and waist–hip ratio, may have affected the accuracy of the groups formed according to BMI. Lack of registration of IV fluids and diuretic treatments given before the ICU follow-up may have affected BMI and AKI data by changing the weight and urine amount measured in the ICU. Also, the use of diuretic therapy during follow-up in the ICU may have changed the AKI stage of the patients.

In conclusion, it was determined that the risk of developing AKI was higher in obese patients, but not in those who are in serious conditions. Another paradox was that the development of AKI was associated with a higher mortality rate in normal-weight and overweight patients, but not in obese patients. In addition, cerebrovascular diseases pose an additional risk of AKI as a reason for hospitalization. The lack of difference in mortality between patients with and without AKI supports the obesity paradox in the ICU. However, further research in a larger cohort is required to better determine the effect of obesity in the ICU. Doing so may help identify patients who would most likely benefit from early AKI prevention and treatment.

## References

[1] Wang S, Liu X, Chen Q, et al. The role of increased body mass index in outcomes of sepsis: a systematic review and meta-analysis. BMC Anesthesiol. 2017;17(1):118. 28859605 10.1186/s12871-017-0405-4 PMC5579888

[2] Flegal KM, Carroll MD, Kuczmarski RJ, et al. Overweight and obesity in the United States: prevalence and trends, 1960–1994. Int J Obes Relat Metab Disord. 1998;22(1):39–47. 9481598 10.1038/sj.ijo.0800541

[3] Flegal KM, Carroll MD, Ogden CL, et al. Prevalence and trends in obesity among US adults, 1999–2008. JAMA. 2010;303(3):235–241. 20071471 10.1001/jama.2009.2014

[4] Macha M, Molina EJ, Franco M, et al. Pre-transplant obesity in heart transplantation: are there predictors of worse outcomes? Scand Cardiovasc J. 2009;43(5):304–310. 19291586 10.1080/14017430902810911

[5] Schetz M, De Jong A, Deane AM, et al. Obesity in the critically ill: a narrative review. Intensive Care Med. 2019;45(6):757–769. 30888440 10.1007/s00134-019-05594-1

[6] Robinson MK, Mogensen KM, Casey JD, et al. The relationship among obesity, nutritional status, and mortality in the critically ill. Crit Care Med. 2015;43(1):87–100. 25289931 10.1097/CCM.0000000000000602

[7] Sakr Y, Madl C, Filipescu D, et al. Obesity is associated with increased morbidity but not mortality in critically ill patients. Intensive Care Med. 2008;34(11):1999–2009. 18670756 10.1007/s00134-008-1243-0

[8] Shashaty MG, Stapleton RD. Physiological and management implications of obesity in critical illness. Ann Am Thorac Soc. 2014;11(8):1286–1297. 25172506 10.1513/AnnalsATS.201404-159FR PMC4298999

[9] Wacharasint P, Fuengfoo P, Rangsin R, et al. Prevalence and impact of overweight and obesity in critically ill surgical patients: analysis of THAI-SICU study. J Med Assoc Thai. 2016;99(6):55–62. 29906082

[10] Joffe A, Wood K. Obesity in critical care. Curr Opin Anaesthesiol. 2007;20(2):113–118. 17413393 10.1097/ACO.0b013e3280803d5f

[11] Nasraway SA Jr, Albert M, Donnelly AM, et al. Morbid obesity is an independent determinant of death among surgical critically ill patients. Crit Care Med. 2006;34(4):964–970. 16484910 10.1097/01.CCM.0000205758.18891.70

[12] Goulenok C, Monchi M, Chiche JD, et al. Influence of overweight on ICU mortality: a prospective study. Chest. 2004;125(4):1441–1445. 15078757 10.1378/chest.125.4.1441

[13] Bercault N, Boulain T, Kuteifan K, et al. Obesity-related excess mortality rate in an adult intensive care unit: a risk-adjusted matched cohort study. Crit Care Med. 2004;32:998–1003. 15071392 10.1097/01.ccm.0000119422.93413.08

[14] Akinnusi ME, Pineda LA, El Solh AA. Effect of obesity on intensive care morbidity and mortality: a meta-analysis. Crit Care Med. 2008;36:151–158. 18007266 10.1097/01.CCM.0000297885.60037.6E

[15] Oliveros H, Villamor E. Obesity and mortality in critically ill adults: a systematic review and meta-analysis. Obesity (Silver Spring). 2008;16(3):515–521. 18239602 10.1038/oby.2007.102

[16] Ni YN, Luo J, Yu H, et al. Can body mass index predict clinical outcomes for patients with acute lung injury/acute respiratory distress syndrome? Crit Care. 2017;21(1):36. 28222804 10.1186/s13054-017-1615-3 PMC5320793

[17] Schiffl H. Obesity and the survival of critically ill patients with acute kidney injury: a paradox within the paradox? Kidney Dis. 2020;6(1):13–21. 10.1159/000502209 PMC6995946 32021870

[18] Druml W, Metnitz B, Schaden E, et al. Impact of body mass on incidence and prognosis of acute kidney injury requiring renal replacement therapy. Intensive Care Med. 2010;36(7):1221–1228. 20232041 10.1007/s00134-010-1844-2

[19] Chao CT, Wu VC, Tsai HB, et al. Impact of body mass on outcomes of geriatric postoperative acute kidney injury patients. Shock. 2014;41(5):400–405. 25133600 10.1097/SHK.0000000000000143

[20] Kim H, Kim H, Lee M, et al. The impact of disease severity on paradoxical association between body mass index and mortality in patients with acute kidney injury undergoing continuous renal replacement therapy. BMC Nephrol. 2018;19(1):32. 29415663 10.1186/s12882-018-0833-5 PMC5804063

[21] Pedersen AB, Gammelager H, Kahlert J, et al. Impact of body mass index on risk of acute kidney injury and mortality in elderly patients undergoing hip fracture surgery. Osteoporos Int. 2017;28(3):1087–1097. 27866215 10.1007/s00198-016-3836-8

[22] Soto GJ, Frank AJ, Christiani DC, et al. Body mass index and acute kidney injury in the acute respiratory distress syndrome. Crit Care Med. 2012;40(9):2601–2608. 22732288 10.1097/CCM.0b013e3182591ed9 PMC3423468

[23] Danziger J, Chen KP, Lee J, et al. Obesity, acute kidney injury, and mortality in critical illness. Crit Care Med. 2016;44(2):328–334. 26496453 10.1097/CCM.0000000000001398 PMC4715729

[24] Gameiro J, Gonçalves M, Pereira M, et al. Obesity, acute kidney injury and mortality in patients with sepsis: a cohort analysis. Ren Fail. 2018;40(1):120–126. 29388454 10.1080/0886022X.2018.1430588 PMC6014496

[25] Kidney International Supplements. 2012. 2, 1; 10.1038/kisup.2012.2 PMC4089660 25028631

[26] World Health Organization: Body mass index – BMI. [cited 2020 Aug 20]. Available from: http://www.euro.who.int/en/health-topics/disease-prevention/nutrition/a-healthy-lifestyle/body-mass-index-bmi.

[27] Yap CH, Mohajeri M, Yii M. Obesity and early complications after cardiac surgery. Med J Aust. 2007;186(7):350–354. 17407431 10.5694/j.1326-5377.2007.tb00935.x

[28] Chagnac A, Weinstein T, Korzets A, et al. Glomerular hemodynamics in severe obesity. Am J Physiol Renal Physiol. 2000;278(5):F817–F822. 10807594 10.1152/ajprenal.2000.278.5.F817

[29] Chagnac A, Weinstein T, Herman M, et al. The effects of weight loss on renal function in patients with severe obesity. J Am Soc Nephrol. 2003;14(6):1480–1486. 12761248 10.1097/01.asn.0000068462.38661.89

[30] Cheng CF, Lian WS, Chen SH, et al. Protective effects of adiponectin against renal ischemia-reperfusion injury via prostacyclin-PPARα-heme oxygenase-1 signaling pathway . J Cell Physiol. 2012;227(1):239–249. 21412771 10.1002/jcp.22726

[31] Han DC, Isono M, Chen S, et al. Leptin stimulates type I collagen production in db/db mesangial cells: glucose uptake and TGF-beta type II receptor expression. Kidney Int. 2001;59(4):1315–1323. 11260392 10.1046/j.1523-1755.2001.0590041315.x

[32] Nakamura K, Fuster JJ, Walsh K. Adipokines: a link between obesity and cardiovascular disease. J Cardiol. 2014;63(4):250–259. 24355497 10.1016/j.jjcc.2013.11.006 PMC3989503

[33] Ng PY, Eikermann M. The obesity conundrum in sepsis. BMC Anesthesiol. 2017;17(1):147. 29070011 10.1186/s12871-017-0434-z PMC5657099

[34] Yamahara K, Kume S, Koya D. Obesity-mediated autophagy insufficiency exacerbates proteinuria-induced tubulointerstitial lesions. J Am Soc Nephrol. 24(11):1769–1781. 10.1681/ASN.2012111080 PMC3810079 24092929

[35] Mullens W, Abrahams Z, Francis GS, et al. Importance of venous congestion for worsening of renal function in advanced decompensated heart failure. J Am Coll Cardiol. 2009;53(7):589–596. 19215833 10.1016/j.jacc.2008.05.068 PMC2856960

[36] Kim IB, Prowle J, Baldwin I, et al. Incidence, risk factors and outcome associations of intra-abdominal hypertension in critically ill patients. Anaesth Intensive Care. 2012;40(1):79–89. 22313065 10.1177/0310057X1204000107

[37] Glance LG, Wissler R, Mukamel DB, et al. Perioperative outcomes among patients with the modified metabolic syndrome who are undergoing noncardiac surgery. Anesthesiology. 2010;113(4):859–852. 20808207 10.1097/ALN.0b013e3181eff32e

[38] Duane TM, Dechert T, Aboutanos MB, et al. Obesity and outcomes after blunt trauma. J Trauma. 2006;61:1218–1221. 17099532 10.1097/01.ta.0000241022.43088.e1

[39] Varon J, Marik P. Management of the obese critically ill patient. Crit Care Clin. 2001;17(1):187–200. 11219229 10.1016/s0749-0704(05)70159-7

[40] De Jong A, Wrigge H, Hedenstierna G, et al. How to ventilate obese patients in the ICU. Intensive Care Med. 2020;46(12):2423–2435. 33095284 10.1007/s00134-020-06286-x PMC7582031

[41] Lee CK, Tefera E, Colice G. The effect of obesity on outcomes in mechanically ventilated patients in a medical intensive care unit. Respiration. 2014;87(3):219–226. 24457313 10.1159/000357317

[42] O’Brien JM, Philips GS, Ali NA, et al. The association between body mass index, processes of care, and outcomes from mechanical ventilation: a prospective cohort study. Crit Care Med. 2012;40(5):1456–1463. 22430246 10.1097/CCM.0b013e31823e9a80

[43] Pepin J, Borel JC, Janssens JP. Obesity hypoventilation syndrome: an underdiagnosed and undertreated condition . Am J Respir Crit Care Med. 2012;186(12):1205–1207. 23250497 10.1164/rccm.201210-1922ED

[44] Chlif M, Keochkerian D, Choquet D, et al. Effects of obesity on breathing pattern, ventilatory neural drive and mechanics. Respir Physiol Neurobiol. 2009;168(3):198–202. 19559105 10.1016/j.resp.2009.06.012

[45] Salome CM, King GG, Berend N. Physiology of obesity and effects on lung function. J Appl Physiol (1985). 2010;108(1):206–211. 19875713 10.1152/japplphysiol.00694.2009

[46] Pelosi P, Croci M, Ravagnan I, et al. Total respiratory system, lung, and chest wall mechanics in sedated-paralyzed postoperative morbidly obese patients. Chest. 1996;109(1):144–151. 8549177 10.1378/chest.109.1.144

[47] Kress JP, Pohlman AS, Alverdy J, et al. The impact of morbid obesity on oxygen cost of breathing (VO(2RESP)) at rest. Am J Respir Crit Care Med. 1999;160(3):883–886. 10471613 10.1164/ajrccm.160.3.9902058

[48] De Jong A, Chanques G, Jaber S. Mechanical ventilation in obese ICU patients: from intubation to extubation. Crit Care. 2017;21(1):63. 28320439 10.1186/s13054-017-1641-1 PMC5359820

[49] Sakr Y, Elia C, Mascia L, et al. Being overweight or obese is associated with decreased mortality in critically ill patients: a retrospective analysis of a large regional Italian multicenter cohort. J Crit Care. 2012;27(6):714–721. 23102526 10.1016/j.jcrc.2012.08.013

[50] Gong MN, Bajwa EK, Thompson BT, et al. Body mass index is associated with the development of acute respiratory distress syndrome. Thorax. 2010;65(1):44–50. 19770169 10.1136/thx.2009.117572 PMC3090260

[51] Ju S, Lee TW, Yoo JW, et al. Body Mass Index as a predictor of acute kidney injury in critically ill patients: a retrospective single-center study. Tuberc Respir Dis. 2018;81(4):311–318. 10.4046/trd.2017.0081 PMC6148097 29926539

[52] Winkelman C, Maloney B, Kloos J. The impact of obesity on critical care resource use and outcomes. Crit Care Nurs Clin North Am. 2009;21(3):403–422. 19840718 10.1016/j.ccell.2009.07.002

[53] Pepper DJ, Sun J, Welsh J, et al. Increased body mass index and adjusted mortality in ICU patients with sepsis or septic shock: a systematic review and meta-analysis. Crit Care. 2016;20(1):181. 27306751 10.1186/s13054-016-1360-z PMC4908772

[54] Tocalini P, Vicente A, Amoza RL, et al. Asociación entre obesidad y mortalidad en pacientes adultos que reciben ventilación mecánica invasiva: una revisión sistemática y metaanálisis. Med Intens; 2018;44:18–26. 10.1016/j.medin.2018.07.006 30195445

[55] Wang H, Shi Y, Bai Z-H, et al. Higher body mass index is not a protective risk factor for 28-days mortality in critically ill patients with acute kidney injury undergoing continuous renal replacement therapy. Ren Fail. 2019;41(1):726–732. 31424314 10.1080/0886022X.2019.1650767 PMC6713092

[56] Falagas ME, Athanasoulia AP, Peppas G, et al. Effect of body mass index on the outcome of infections: a systematic review. Obes Rev. 2009;10(3):280–289. 19243518 10.1111/j.1467-789X.2008.00546.x

[57] Kim H, Kim J, Seo C, et al. Body mass index is inversely associated with mortality in patients with acute kidney injury undergoing continuous renal replacement therapy. Kidney Res Clin Pract. 2017;36(1):39–47. 28392996 10.23876/j.krcp.2017.36.1.39 PMC5331974

[58] Deng F, Peng M, Li J, et al. Nomogram to predict the risk of septic acute kidney injury in the first 24 h of admission: an analysis of intensive care unit data. Ren Fail. 2020;42(1):428–436. 32401139 10.1080/0886022X.2020.1761832 PMC7269058

